# Sporadic Multiple Meningiomas Presenting With Drug-Resistant Seizures in a Young Adult: A Case Report

**DOI:** 10.7759/cureus.116062

**Published:** 2026-09-10

**Authors:** Meryem Amghar, Zakariae Benyaich, Mohamed Lmejjati

**Affiliations:** 1 Department of Neurosurgery, Centre Hospitalier Universitaire (CHU) Mohammed VI, Ibn Zohr University, Agadir, MAR

**Keywords:** chronic disease, drug-resistant seizures, multiple meningiomas, recurrence, subtotal resection

## Abstract

Meningiomas represent the most common benign primary intracranial tumors. However, multiple meningiomas (MMs) are relatively rare and can be challenging to manage, often requiring repeated surgical interventions and long-term follow-up. Here, we present the case of a 28-year-old woman who was operated on in 2019 for a right parasagittal MM, WHO grade 1. Her initial presentation was characterized by seizures and left-sided hemiplegia. Seven years later, she presented with recurrent seizures that were resistant to medical treatment. Neurological examination revealed residual left-sided spastic pyramidal syndrome. Brain MRI revealed multiple extra-axial dural-based lesions, including a large right frontal parasagittal mass. Subtotal resection of the largest lesion was performed. Histopathology confirmed the diagnosis of WHO grade 1 mixed meningothelial and fibroblastic MM. The postoperative course was uneventful. This case highlights the challenges associated with sporadic MMs, particularly long-term surveillance, tumor recurrence, and the potential need for repeated surgical interventions.

## Introduction

Meningiomas are the most common primary intracranial tumors in adults, accounting for approximately 38% of all brain tumors and 55% of non-malignant primary ones [1]. They typically present as solitary, slow-growing lesions, with an overall good prognosis after complete resection. They are characterized by 15 histological subtypes [2]. However, meningiomas (MMs) are defined as two or more spatially separated synchronous or metachronous lesions; they represent a distinct and much rarer entity, accounting for an estimated 1%-10% of all MM cases [1]. Unlike solitary tumors, MM tends to behave as a chronic disease. Consequently, disease control, rather than cure, is often the realistic goal, and patients may face repeated interventions and lifelong radiological follow-up [1].

It is noteworthy that sporadic MMs are exceptionally rare in young adults, as they typically occur in the sixth decade of life, making their presentation in a 28-year-old patient particularly unusual.

Seizures are among the most clinically significant manifestations of supratentorial MMs, particularly those involving the convexity or parasagittal region, and can persist even after surgical treatment [1,3]. When seizures become refractory to antiseizure medication, the interplay between tumor recurrence, cortical irritation, and the residual effects of prior surgery complicates both diagnosis and management [3]. 

We report the case of a young woman with a history of a resected parasagittal MM who developed multiple recurrent dural-based lesions revealed by drug-resistant epilepsy, illustrating the diagnostic and therapeutic challenges posed by this rare disease pattern.

## Case presentation

A 28-year-old woman with no family history of MM and no history of radiation exposure was admitted to our neurosurgical department in 2026 for recurrent convulsive seizures that had become resistant to medical treatment.

Her relevant medical history dates back to 2019, when she underwent surgery for a right parasagittal MM, classified as WHO grade 1. At that time, she presented with focal seizures progressing to bilateral tonic-clonic activity associated with left-sided hemiplegia. Following surgery, antiepileptic therapy was initiated with sodium valproate (1500 mg/day) and lacosamide (200 mg/day).

In March 2026, the patient experienced recurrent convulsions, despite ongoing antiepileptic therapy, in addition to moderate headache, with no vomiting or new focal neurological deficit. The recurrence of seizures despite antiseizure medication prompted further radiological and neurosurgical evaluations.

On admission, the Karnofsky Performance Status was 70%. The patient was conscious, with a Glasgow Coma Scale score of 15/15 and no signs of acute vital distress. Neurological examination revealed a left-sided pyramidal syndrome with residual spasticity and muscle strength graded 4/5.

Brain MRI revealed multiple intensely enhancing extra-axial dural-based lesions predominantly involving the parasagittal and convexity regions in both hemispheres. A dominant right frontal parasagittal lesion was identified, along with several additional enhancing lesions, supporting the diagnosis of MMs (Figure 1).

**Figure 1:**
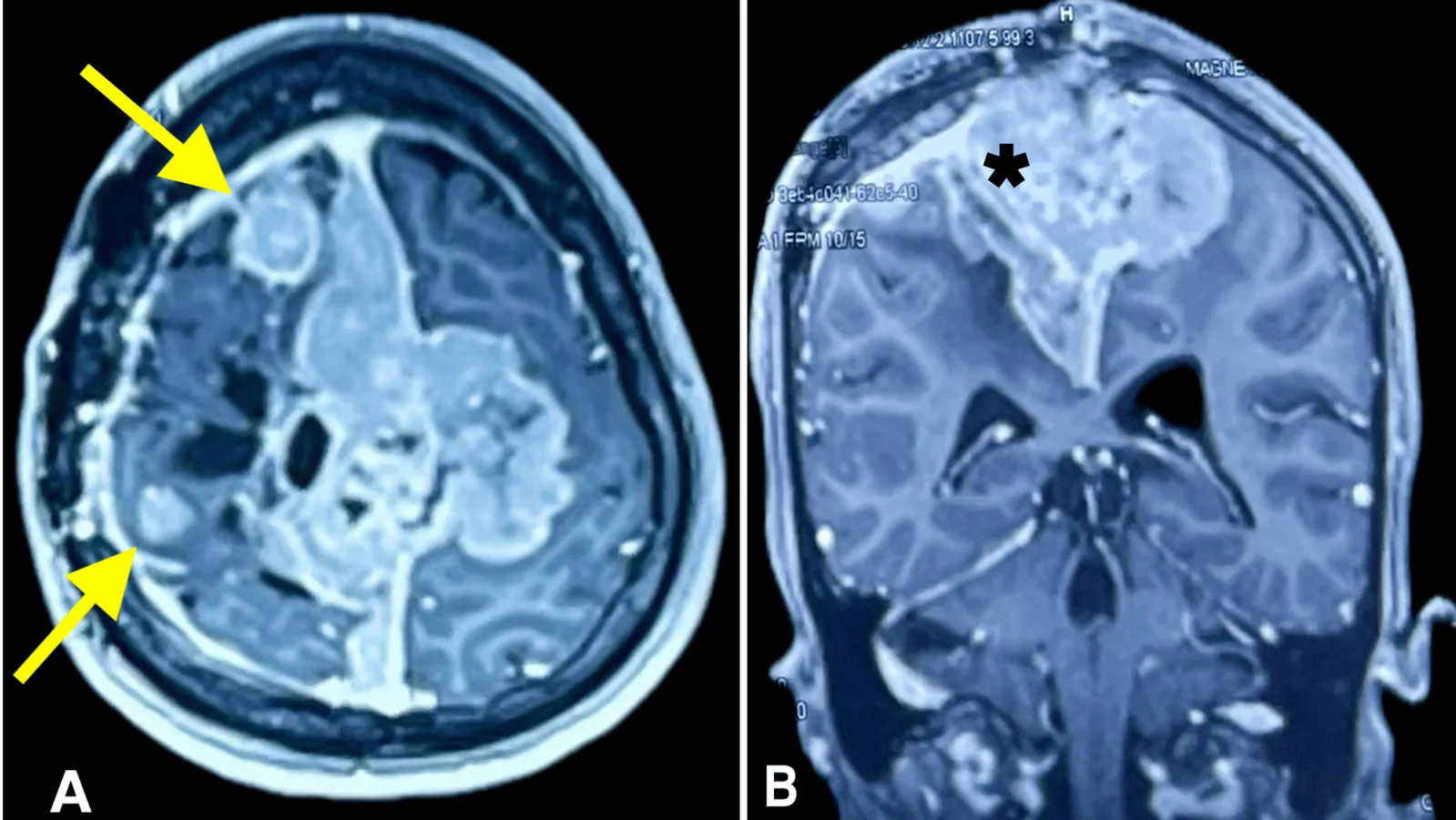
Preoperative axial (A) and coronal (B) contrast-enhanced T1-weighted MRI images showing multiple extra-axial, dural-based, strongly enhancing lesions (yellow arrows), including a dominant right frontal parasagittal mass (asterisk).

The patient had no clinical or familial features suggestive of a known tumor predisposition syndrome.

Given the recurrence of clinically significant seizures and the presence of a dominant right frontal parasagittal lesion, the patient underwent a surgical subtotal resection of that largest mass. 

The intervention was based on a right parasagittal craniotomy, and the tumor was surgically debulked with careful dissection from the adjacent cortical structures (Figure 2). Given its close relationship with the superior sagittal sinus, complete resection was not feasible. A Simpson grade IV resection was therefore achieved, with residual tumor intentionally left in situ to avoid sinus injury.

**Figure 2:**
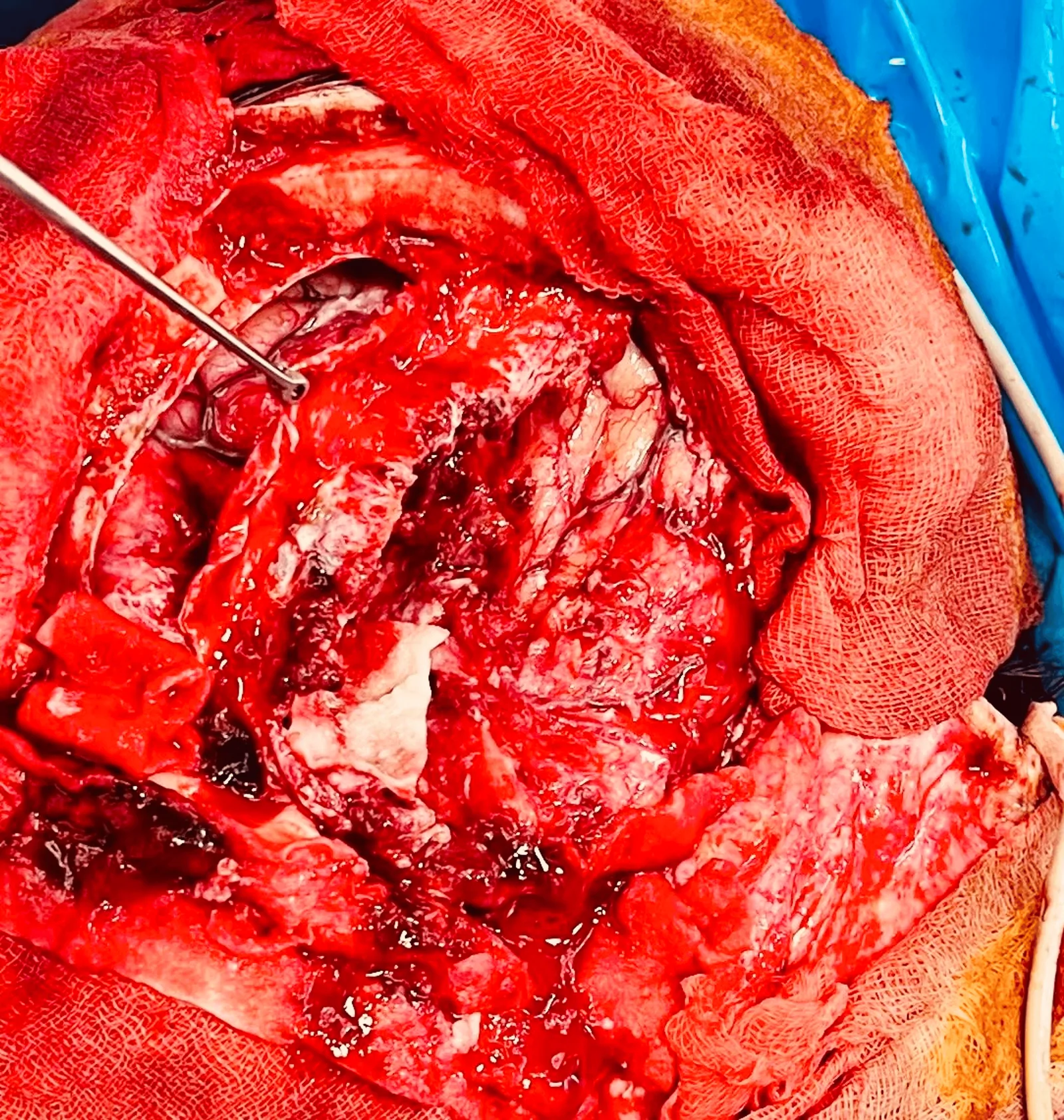
Perioperative image of the right parasagittal lesion subtotal resection

The postoperative course was uncomplicated, with no significant perioperative or postoperative adverse events. The patient was monitored in the intensive care unit for 48 hours before being transferred to the neurosurgical department.

Histopathological examination revealed a mixed MM comprising meningothelial and fibroblastic components. The tumor was classified as WHO grade 1 according to the fifth edition of the WHO Classification of Tumors of the Central Nervous System (2021), with no histological features of malignancy [2]. Although the Ki-67 labeling index is an important prognostic marker in MMs, Ki-67 immunohistochemical assessment was not available in our pathology laboratory; therefore, it could not be performed in this patient.

An early postoperative contrast-enhanced brain MRI was obtained five days after the intervention. It demonstrated a right frontal parasagittal postoperative resection cavity with residual enhancing tissue along the parasagittal dura, consistent with subtotal resection. Multiple additional intensely enhancing extra-axial lesions remained visible (Figure 3). 

**Figure 3:**
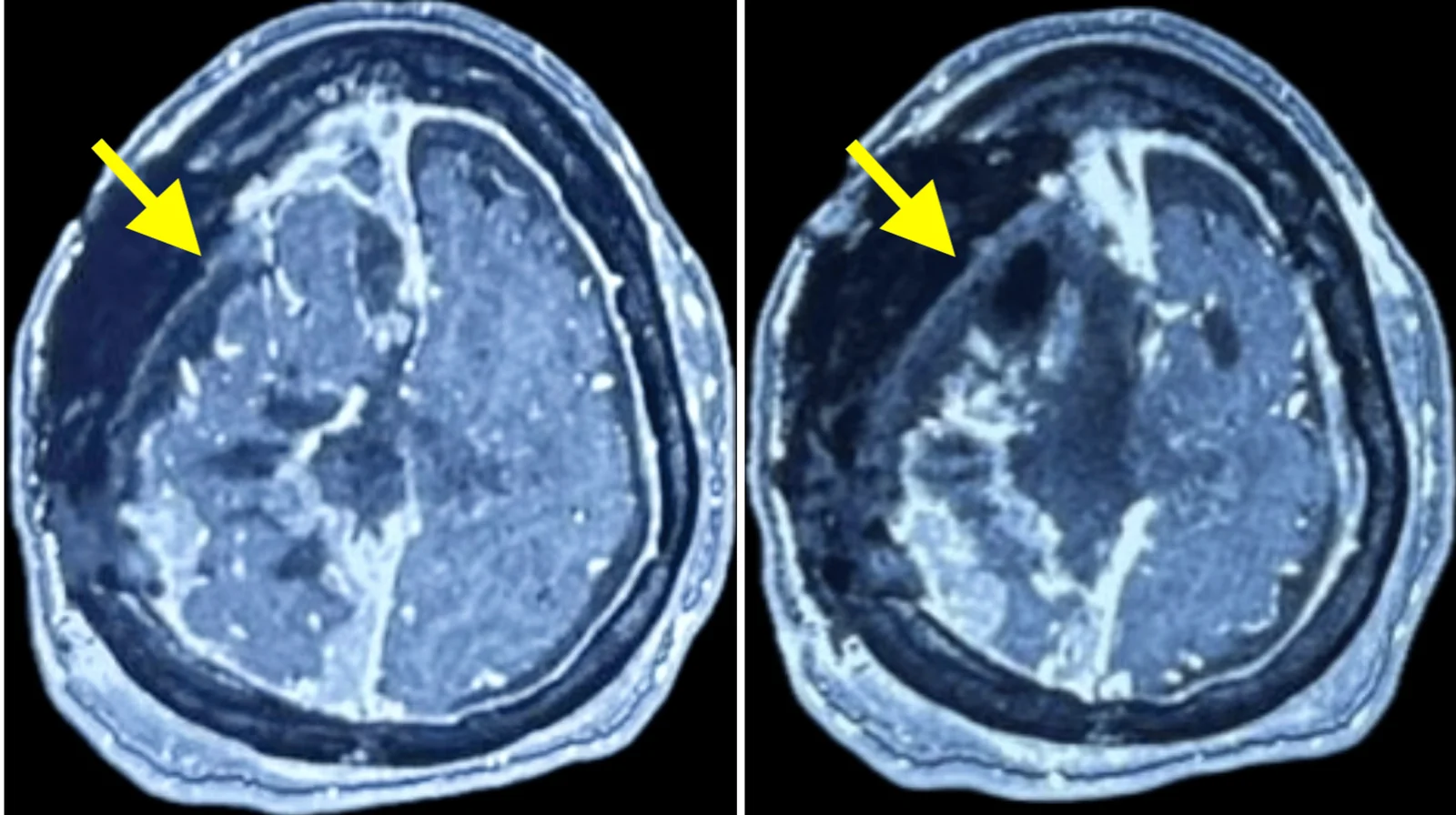
Axial contrast-enhanced T1-weighted MRI illustrating a right frontal parasagittal postoperative resection cavity (yellow arrows) with residual enhancing tissue.

No postoperative seizures were observed during the follow-up period. The same antiseizure therapy was continued with sodium valproate and lacosamide. The patient remained clinically stable, with no newly documented neurological deficits, and was discharged after an uncomplicated postoperative recovery.

## Discussion

MMs are less frequent than solitary ones, with the first case reported in the literature in 1889. They can be sporadic, radiation-induced, or associated with hereditary syndromes, such as neurofibromatosis type 2, Cowden syndrome, Li Fraumeni syndrome, Gorlin syndrome, or Werner Syndrome [1,4].

MM’s pathophysiology remains unclear, with two hypotheses having been proposed. The first suggests that each tumor develops independently. The second proposes that all lesions originate from a single abnormal cell clone that spreads through the cerebrospinal fluid [1,5].

MMs represent the most prevalent neoplasms induced by radiation [1,5]. Hence, in the absence of both personal and familial medical history, sporadic MM remains a very uncommon entity, as observed in our patient.

MMs most commonly present during the sixth decade of life with female predominance [5,6], which is the same as for patients with a single MM.

According to several large series, sporadic MM most commonly manifests either as multiple distinct lesions at the time of presentation, with a median of two separate lesions, or as a solitary MM initially, followed by the development of additional lesions during follow-up [5,6]. At diagnosis, a substantial proportion of patients are asymptomatic, with MMs discovered incidentally on neuroimaging performed for other indications [1,6]. 

In the present case, however, the disease course was symptomatic from the beginning, with seizures and a focal motor deficit dominating the clinical picture at both the initial 2019 presentation and the 2026 recurrence.

Overall, the clinical manifestation of MM is related to the lesions' size, their location, and peritumoral edema.

The most common locations of MM are the cerebral convexities, followed by the skull base [1,6]. In addition, the lesions are generally small, measuring 2 cm or less. This may explain the high incidence of asymptomatic cases [5,6].

The radiological appearance of multiple intensely enhancing, extra-axial, dural-based lesions can be challenging given that it is not specific to MM. Meningeal metastases, most often from breast or lung carcinoma, are the leading differential diagnosis, as they can mimic MMs radiologically. Other considerations include dural infiltration by lymphoma, leukemia, sarcoidosis, IgG4-related disease, tuberculous pachymeningitis, and, more rarely, solitary fibrous tumor. Clinical history, systemic evaluation, and histopathological confirmation are therefore essential for establishing the right diagnosis [7].

The main challenge in front of a patient with MM remains the management decision, which is more complex than that of solitary MMs. Because complete eradication of every lesion is frequently neither feasible nor necessary, treatment decisions are individualized around the tumor causing the greatest clinical burden [5,6], as reflected in this case by the selective resection of the dominant, presumed epileptogenic, right frontal parasagittal lesion rather than an attempt to address all visible lesions simultaneously. This symptomatic lesion approach is consistent with published management paradigms for MM, which favor surgery or radiosurgery for growing or symptomatic tumors while reserving observation with serial MRI for smaller, stable, asymptomatic lesions [1].

Seizures in patients with MM can be explained by cortical irritation of the tumor itself or by the surrounding gliotic changes induced by prior surgery. Hence, they are more frequent with convexity and parasagittal locations than with skull base tumors [3,8]. Postoperative seizure control does not always follow resection, and a subset of patients -- as in the present case -- continue to experience seizures that are resistant to standard antiseizure medications [8,9]. 

In our patient, we believe that the recurrence of seizures was more likely related to the progressive growth of the recurrent right frontal lesion, resulting in cortical irritation. Chronic postoperative gliosis related to the 2019 surgery may also have contributed to the epileptogenic substrate; however, the temporal association between tumor recurrence and seizure recurrence, together with the improvement in seizure control following tumor resection, supports tumor progression as the predominant contributing factor.

Subtotal resection, chosen here to limit the risk of new motor deficit given the lesion's proximity to the sagittal sinus and motor cortex, still achieves meaningful cytoreduction and can contribute to seizure control, even though it leaves residual tumor requiring ongoing surveillance and discussion of fractionated radiotherapy or stereotactic radiosurgery [1].

Although radiotherapy may provide effective local control, its potential long-term risks, including malignant transformation, are particularly relevant given the patient's young age and long expected lifespan. Therefore, the decision to irradiate residual disease should be individualized, balancing the risk of tumor progression against the potential late effects of radiation.

The histological finding of a mixed meningothelial and fibroblastic WHO grade 1 MM in our patient is consistent with previous reports indicating that MMs share similar histological characteristics with solitary MMs. The most frequently reported subtypes include meningothelial, fibroblastic, transitional, and psammomatous MMs [1,2]. Although WHO grade 1 histology indicates the benign features of the tumor, it does not exclude the risk of recurrence or the development of new lesions. Therefore, long-term radiological surveillance remains essential, even after successful surgical resection.

According to the European Association for Neuro-Oncology (EANO) guidelines, annual MRI is recommended for patients with WHO grade 1 MMs during the first five years, after which the imaging interval may be extended based on the clinical and radiological course [1].

This case adds to the limited literature on young patients with recurrent MMs presenting with pharmacoresistant epilepsy, and it illustrates the value of a lesion-targeted surgical strategy combined with sustained multidisciplinary follow-up in patients for whom cure is unlikely, but durable disease and symptom control remain realistic goals [5,6].

## Conclusions

MM is considered a chronic disease that can significantly impair the quality of life of young patients, although it is predominantly benign histologically. A pragmatic approach to managing this condition involves individualized, lesion-targeted surgery combined with adequate medical treatments, including antiseizure therapy, radiotherapy, and long-term MRI monitoring of residual lesions. This approach makes it possible to control the severity of symptoms in patients in whom complete disease eradication is not feasible.

In our case, the surgical intervention led to a favorable early clinical outcome, with improvement in seizure control and no new neurological deficits during follow-up. These findings highlight the value of surgical planning in patients with MMs and drug-resistant seizures. More studies are required in this area to define optimal surgical timing, the extent of resection, the role of radiotherapy, and indications for antiseizure medications.
